# Subjective Salience of Birdsong and Insect Song with Equal Sound Pressure Level and Loudness

**DOI:** 10.3390/ijerph17238858

**Published:** 2020-11-28

**Authors:** Yoshiharu Soeta, Ayaka Ariki

**Affiliations:** Biomedical Research Institute, National Institute of Advanced Industrial Science and Technology (AIST), Osaka 563-8577, Japan; ariki.yxuric@aist.go.jp

**Keywords:** soundscape, salience, birdsong, insect song

## Abstract

Birdsong is used to communicate the position of stairwells to visually impaired people in train stations in Japan. However, more than 40% of visually impaired people reported that such sounds were difficult to identify. Train companies seek to present the sounds at a sound pressure level that is loud enough to be detected, but not so loud as to be annoying. Therefore, salient birdsongs with relatively low sound pressure levels are required. In the current study, we examined the salience of different types of birdsong and insect song, and determined the dominant physical parameters related to salience. We considered insect songs because both birdsongs and insect songs have been found to have positive effects on soundscapes. We evaluated subjective saliences of birdsongs and insect songs using paired comparison methods, and examined the relationships between subjective salience and physical parameters. In total, 62 participants evaluated 18 types of bird songs and 16 types of insect sounds. The results indicated that the following features significantly influenced subjective salience: the maximum peak amplitude of the autocorrelation function, which signifies pitch strength; the interaural cross-correlation coefficient, which signifies apparent source width; the amplitude fluctuation component; and spectral content, such as flux and skewness.

## 1. Introduction

Birdsong and insect song are near universal experiences in the outdoor environment. Although some birdsongs and insect songs communicate seasonal changes and are considered pleasant by Japanese listeners, not all instances of birdsong elicit pleasant feelings [1]. Further, while not all birdsongs and insect songs are considered by humans to be beneficial components of an environment, some have been found to contribute to perceived attention restoration and stress recovery [2,3].

From the perspective of soundscapes, natural sounds (e.g., water, birdsongs, and wind in trees) can play a key role in acoustic comfort. Water sounds are often used to mask other sounds and as noise barriers to enhance urban soundscapes [4,5,6]. The introduction of birdsong has been found to increase the subjective pleasantness of soundscapes in public spaces [7]. Among various natural sounds, birdsong was judged as the most effective and beneficial type of sound for improving sound environments [8,9,10].

In Japanese public spaces, sound signals are often used to guide visually impaired people to specific destinations, such as a ticket gate or staircase. For instance, birdsong is often used to signal the presence of a staircase in train stations. However, more than 40% of visually impaired people reported that a birdsong stimulus was difficult to localize in a train station setting [11]. Although guidelines exist regarding the use of birdsongs as information signals [12], these are not always strictly followed by train company staff, who may prefer to use lower-than-recommended sound pressure levels (SPLs) to reduce discomfort in customers, staff, and surrounding residents.

The physical factors that affect the sound signals used to guide visually impaired people have been investigated from the viewpoint of sound localization. Based on the percentages of correct localization responses, researchers have found that the signal to noise ratio, initial delay time, reverberation energy, distance, elevation angle, and the temporal pattern of the signal all affect sound localization [13,14]. Additionally, researchers have proposed that sounds with specific temporal patterns, such as those with particular early component and silent interval lengths, might be more easily detectable by visually impaired people, and further, that these factors might be uncovered by examining human brain responses [15].

The aim of this study was to evaluate the salience of a number of birdsongs and insect songs, and to determine the physical factors that modulated the observed salience. Here, we used the term salience to refer to whether or not a sound stood out from background noise. While previous studies have indicated that loudness is a significant predictor of salience [16,17], it is preferable that sound signals used for guidance purposes be salient even at a low SPL, as this reduces unnecessary discomfort elicited by loud sounds in the environment. Although the abovementioned guidelines include an appropriate volume in SPL, they do not consider variations among specific sound sources [12]. To address this in the present study, we investigated the subjective salience of birdsong and insect song presented with equal SPLs and loudness to clarify the effects of physical factors in physically and subjectively equal sound intensity conditions.

## 2. Materials and Methods

### 2.1. Subjective Salience Test

As stimuli, we used 18 types of birdsongs and 16 types of insect songs that had been used in previous experiments [1]. The abovementioned guidelines include some recommendations regarding acoustic specifications [12]. For example, pure tones are not acceptable, sounds with broader frequency bands, frequency fluctuations, amplitude fluctuations, and a duration of less than 5 s are preferable. We selected birdsongs and insect songs that most closely met these specifications. The stimuli were birdsongs produced by Halcyon coromanda (HC), Latham (L), Cuculus canorus (CC), Cuculus saturatus (CS), Strix uralensis (SU), Otus scops (OS), Caprimulgus indicus (CI), Streptopelia orientalis (SO), Terpsiphone atrocaudata (TA), Garrulus glandarius (GG), Porzana fusca (PF), Parus minor (PM), Horornis diphone (HD), Zosterops japonicus (ZJ), Turdus sibiricus (TS), Prunella rubida (PR), Eophona personata (EP), and Emberiza cioides (EC), and insect songs produced by Cryptotympana facialis (CF), Meimuna opalifera (MO), Graptopsaltria nigrofuscata (GN), Oncotympana maculaticollis (OM), Tanna japonensis (TJ), Velarifictorus micado (VM), Loxoblemmus doenitzi (LD), Oecanthus longicauda (OL), Gryllotalpa orientalis (GO), Teleogryllus emma (TE), Meloimorpha japonica (MJ), Xenogryllus marmoratus (XM), Hexacentrus hareyamai (HH), Mecopoda nipponensis (MN), Tettigonia orientalis (TO), and the Japanese katydid (JK).

**Figure 1:**
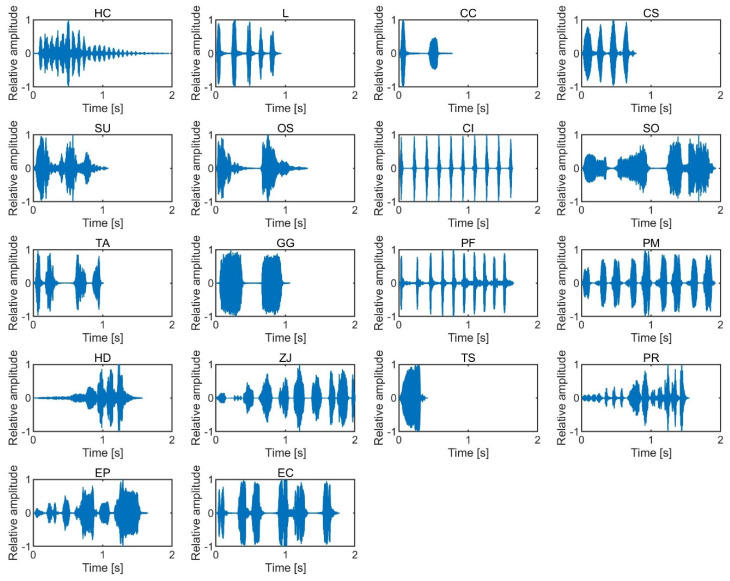
Temporal waveforms of the 18 birdsongs.

**Figure 2:**
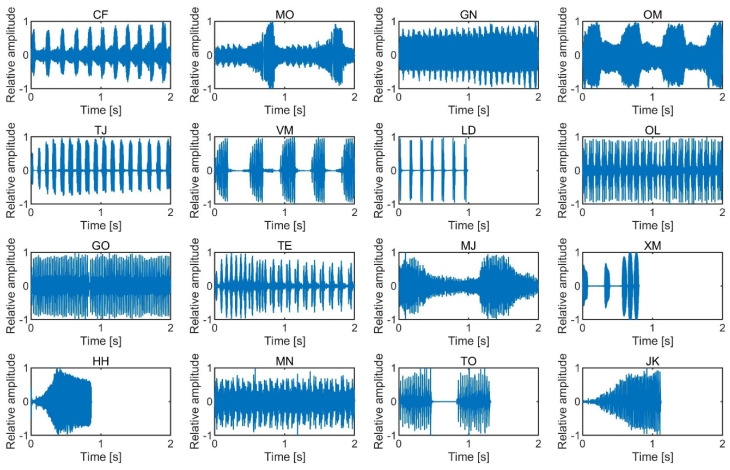
Temporal waveforms of the 16 insect songs.

**Figure 3:**
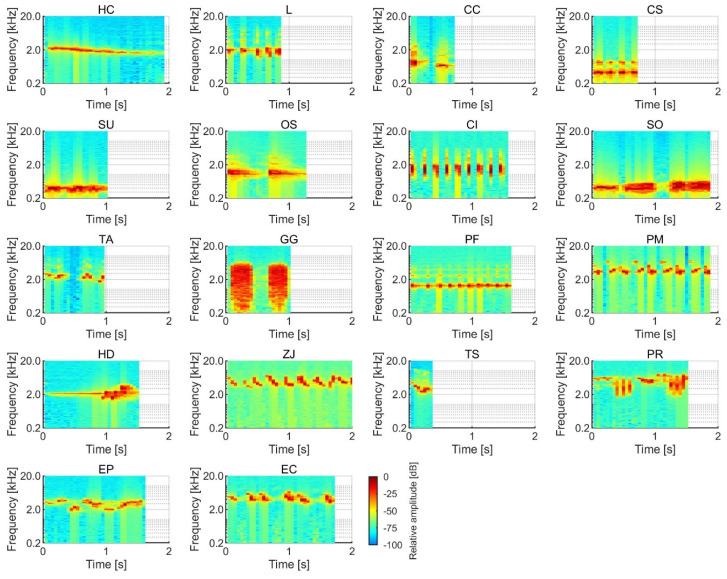
Spectrograms of the 18 birdsongs.

**Figure 4:**
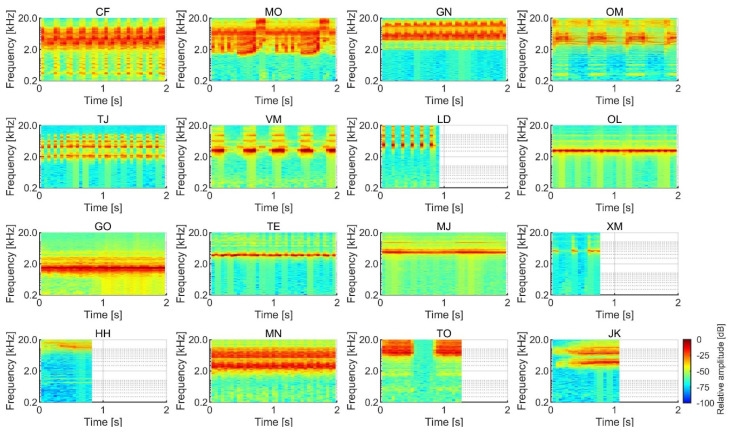
Spectrograms of the 16 insect songs.

The SPLs of the stimuli were analyzed with a temporal window of 5 ms and an interval of 2.5 ms. The stimulus onset and offset were defined as positions with an SPL that was 10 dB higher than the noise floor. We tested subjective salience by presenting birdsong and insect song stimuli with durations between 0.4 and 2.0 s, as the duration is not expected to significantly modulate loudness within that duration range [18]. The temporal waveforms of the birdsongs and insect songs are shown in Figure 1 and Figure 2, and the spectrograms of the sounds are shown in Figure 3 and Figure 4. The stimuli were presented to participants using a headphone amplifier (HDVD800, Sennheiser, Wedemark, Germany) and headphones (HD800, Sennheiser). The participants listened to the stimuli while sitting in a soundproof room with an ambient temperature of 22–25 degrees.

In the equal *L_Aeq_* condition, the birdsongs and insect songs were presented at a continuous A-weighted SPL, i.e., *L_Aeq_*, measured over the duration of each sound of 70 dBA. In the equal loudness condition, the birdsongs and insect songs were presented at 3 sone, which was considered to reflect long-term loudness [19]. The stimuli in the *L_Aeq_* and long-term loudness conditions were verified using a dummy head microphone (KU100, Neumann, Berlin, Germany) and a sound calibrator (Type 4231, Brüel & Kjær, Naerum, Denmark).

In the equal *L_Aeq_* condition, we presented the birdsong and insect song stimuli to 15 participants (11 men) aged between 20 and 41 years (median age of 21.0 years) and 16 participants (11 men) aged between 20 and 41 years (median age of 22.0 years), respectively. We presented both the birdsong and insect songs to 10 participants. In the equal loudness condition, we presented the birdsong and insect song stimuli to 16 participants (seven men) aged between 20 and 54 years (median age of 23.5 years) and 15 participants (seven men) aged between 21 and 54 years (median age of 24.0 years), respectively. We presented both the birdsong and insect songs to 15 participants. We presented the birdsong and insect songs in both the equal *L_Aeq_* and loudness condition to one participant. All participants had normal hearing and no history of neurological disease. According to previous psychoacoustic experiments and our previous studies, we considered the involvement of at least 10 participants to be necessary to ensure sufficient statistical power and the generality of the results [1,20]. Informed consent was obtained from each participant after explaining the nature of the study. The study was approved by the ethics committee of the National Institute of Advanced Industrial Science and Technology (AIST) of Japan (2020–0227).

We used the modified Scheffe’s paired comparison method [21,22,23] to measure subjective salience. In our protocol, we performed all pairwise comparisons for each iteration for each participant. All combinations of pairs (i.e., 153 pairs (N(N − 1)/2, N = 18) for birdsongs and 120 pairs (N(N − 1)/2, N = 16) for insect songs) were presented in random order, and the presentation order within each pair was also randomized. The silent interval between the stimuli was 1.0 s long. Following the presentation of each pair, the participants were asked to judge which stimulus from each pair was more salient using a seven-point scale. Judgments regarding each ordered pair (*i*, *j*) were made using one of the following seven statements: I perceived *i* as strongly more salient than *j* (3 points); I perceived *i* as moderately more salient than *j* (2 points); I perceived *i* as slightly more salient than *j* (1 point); I perceived the salience of the two sounds to be equal (0 points); I perceived *j* as slightly more salient than *i* (−1 point); I perceived *j* as moderately more salient than *i* (−2 points); and, I perceived *j* as strongly more salient than *i* (−3 points). The averaged salience values were calculated based on the modified Scheffe’s method and were defined as scale values (SVs) of salience. An analysis of variance (ANOVA) was conducted on the results of the paired comparison experiments [21,22,23].

### 2.2. Physical Parameters

To quantify the acoustic characteristics of the birdsongs and insect songs, we analyzed specific physical parameters. First, we analyzed three parameters using autocorrelation function (ACF) analysis. The first parameter is the delay time of the first maximum peak, τ_1_, which is related to the perceived pitch. The second parameter is the amplitude of the first maximum peak, ϕ_1_, which is related to the strength of the perceived pitch. The third parameter is the effective duration of the envelope of the normalized ACF, τ_e_, which is defined by the ten-percentile delay and represents a repetitive component in the sound source itself. The fourth parameter is the width of the first decay, W_ϕ(0)_, which is the counterpart to the spectral centroid [24,25].

Based on the results of the interaural cross-correlation function (IACF) analysis, we evaluated three parameters. The first factor was the interaural cross-correlation coefficient (IACC), which was defined by the maximum value of the IACF. The IACC is related to spatial impression, such as subjective diffuseness and apparent source width [26]. The second parameter was the interaural time delay, τ_IACC_, which was defined by the delay time at the IACC. The third parameter was the width of the IACF, W_IACC_, which was defined by the interval of the delay time at a value of 10% below the IACC. The W_IACC_ mainly depends on the frequency component of the signal and is equivalent to the apparent source width [27].

We then analyzed four psychoacoustic parameters: loudness, sharpness, roughness, and fluctuation strength [28]. Loudness is the psychological strength of a sound. Sharpness relates to the balance of the high and low frequency components of a sound. Roughness and fluctuation strength quantify the subjective perception of the rapid (15−300 Hz) and slow (at frequencies up to 20 Hz) amplitude modulation of a sound.

We also analyzed other audio features for sound description, such as the entropy of energy, spectral entropy, spectral flux, and spectral skewness [29]. The entropy of energy is a measure of abrupt changes in the energy level of a sound. Spectral entropy is a measure that is similar to the entropy of energy, but is computed in the frequency domain. Spectral flux measures the speed of spectral change. Spectrum skewness describes the degree of asymmetry in the frequency distribution of a spectrogram of a sound [30].

We analyzed the L_Aeq_, ACF, and IACF parameters. The integration interval was 50 ms and the running step was 1 ms. We also calculated the loudness, sharpness, roughness, fluctuation strength, entropy of energy, spectral entropy, spectral flux, and spectral skewness. The temporal window used for the analysis was 50 ms. The analyses were conducted using a Matlab-based analysis program (Mathworks, Natick, MA, USA).

### 2.3. Multiple Regression Analysis

The normality of each physical parameter was tested using the Shapiro–Wilk test [31]. None of the physical parameters were normally distributed. Because the subjective impression of a sound is influenced not only by average activity, but also by variable components [20,32,33,34,35], we used the median and interquartile range (QR) as predictors of subjective salience. Because sharpness was highly correlated with W_ϕ(0)_ (|r| > 0.79, *p* < 0.01), we excluded it from the multiple regression analysis.

To identify and quantify the physical factors that affect salience, we carried out multiple regression analyses with stepwise selection using a linear combination of the physical factors. The stepping criteria used for entry and removal were based on the significance level of the F-value and were set at 0.05 and 0.10, respectively. Factors with a variance inflation factor of 3.5 or more were excluded to avoid multicollinearity. The analyses were carried out using SPSS statistical analysis software (SPSS version 22.0, IBM Corp., Armonk, NY, USA).

## 3. Results and Discussion

The ANOVA for the SV of salience in the equal *L_Aeq_* condition revealed that the main effect was statistically significant (*F*(17, 4184) = 194.3, *p* < 0.001 for birdsongs; *F*(15, 3479) = 325.6, *p* < 0.001 for insect songs). The ANOVA for the SV of salience in the equal loudness condition revealed that the main effect was statistically significant (*F*(17, 4472) = 187.1, *p* < 0.001 for birdsongs; *F*(15, 3255) = 120.9, *p* < 0.001 for insect songs).

**Figure 5:**
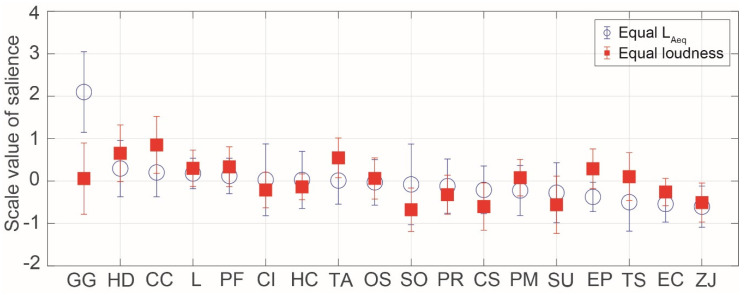
Scale values of saliences for birdsongs in the equal *L_Aeq_* and loudness conditions. The symbols indicate the mean values and the error bars indicate standard deviations.

Figure 5 shows the SVs of salience for birdsongs in the equal *L_Aeq_* and loudness conditions. The most salient birdsong was that of Garrulus glandarius in the equal *L_Aeq_* condition, although the Garrulus glandarius song was not as salient in the loudness condition. This is probably because the *L_Aeq_* of the Garrulus glandarius song was lower than that of the other birdsongs in the equal loudness condition. The relatively salient birdsongs in both the equal *L_Aeq_* and loudness conditions were that of Horornis diphone, Cuculus canorus, Latham, Porzana fusca, and Terpsiphone atrocaudata. Horornis diphone is one of three major species of passeriforme in Japan, which are known for their beautiful vocalizations. Its birdsong was the most preferred stimulus in a previous study [1]. Since ancient times, Cuculus canorus has appeared in various documents in Japan, and its song is often compared to onomatopoeia. Compared with other stimuli, it was found to elicit the largest N1m responses, and these responses were most strongly correlated to the sound envelope in the human brain [15]. The less salient birdsongs in both the equal *L_Aeq_* and loudness conditions were those of Zosterops japonicus, Emberiza cioides, Strix uralensis, and Cuculus saturates.

**Figure 6:**
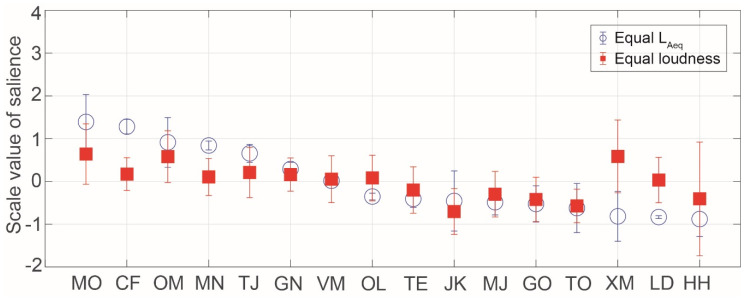
Scale values of saliences for insect songs in the equal *L_Aeq_* and loudness conditions. The symbols indicate the mean values and the error bars indicate standard deviations.

Figure 6 shows the SV of salience for insect songs in the equal *L_Aeq_* and loudness conditions. The most salient insect songs in both the equal *L_Aeq_* and loudness conditions were those produced by Meimuna opalifera, Oncotympana maculaticollis, Mecopoda nipponensis, and Tanna japonensis, which are all cicadas except for Mecopoda nipponensis. Cicadas are famous in Japan as noisy insects in the summertime. The less salient insect songs in both the equal *L_Aeq_* and loudness conditions were those produced by the Japanese katydid, Meloimorpha japonica, Gryllotalpa orientalis, and Tettigonia orientalis. The Japanese katydid and Meloimorpha japonica are well known in Japan and produce sounds during autumn. Regarding the songs of Xenogryllus marmoratus and Hexacentrus hareyamai, the judgment of salience varied among the participants. The songs of Xenogryllus marmoratus and Hexacentrus hareyamai have mainly higher frequency components and shorter durations. This might have caused the large differences between participants.

We conducted a multiple linear regression analysis with the SVs of salience for birdsongs in both the equal *L_Aeq_* and loudness conditions as the outcome variable. The final model showed that IACC, entropy, spectral flux, and spectral skewness were significant parameters in the equal *L_Aeq_* condition, while τ_1_, QR of τ_e_, QR of W_ϕ(0)_, IACC, loudness_QR, and roughness were significant parameters in the equal loudness condition:

*SV*_birdsong in equal SPL_ ≈ b_0_ + b_1_ × IACC+ b_2_ × entropy + b_3_ × spectral flux + b_4_ × spectral skewness, (1)

*SV*_birdsong in equal loudness_ ≈ c_0_ + c_1_ × τ_1_ + c_2_ × τ_e__QR + c_3_ × W_ϕ(0)__QR + c_4_ × IACC + c_5_ × loudness_QR + c_6_ × roughness. (2)

The correlation coefficients between all of the explanatory variables in the equal *L_Aeq_* and loudness conditions are shown in Table 1 and Table 2, respectively. The ANOVA indicated the statistical significance of the model (*F*(5, 264) = 40.34, *p* < 0.001 for the equal *L_Aeq_* condition, *F*(6, 281) = 26.26, *p* < 0.001 for the equal loudness condition). The adjusted coefficient of determination, R^2^, was 0.41 for the equal *L_Aeq_* condition and 0.35 for the equal loudness condition. The standardized partial regression coefficients in Equations (1) and (2) are summarized in Table 3.

The IACC, which signifies the apparent source width, was the significant predictive variable for both the equal *L_Aeq_* and loudness conditions. The partial regression coefficients of the IACC were positive, indicating that birdsongs with a narrower sound source width were perceived as more salient. This is consistent with previous studies regarding the accuracy of sound source localization [36,37]. Spectral flux was also a significant predictive variable in the equal *L_Aeq_* condition, with positive partial regression coefficients. This suggests that quick spectral change led to higher perceived salience.

Higher frequency components might play a key role in saliency. The delay times of the maximum peak amplitude of the ACF, τ_1_, and the spectral skewness were significant predictive variables in the equal loudness and *L_Aeq_* conditions, respectively. The negative τ_1_ regression coefficient indicates that birdsongs with a higher pitch were perceived to be more salient, while the positive regression coefficient of spectral skewness demonstrates that birdsongs with more energy at high frequencies were perceived as more salient.

We also conducted a multiple linear regression analysis for insect songs. The final model showed that the QR of W_ϕ(0)_, QR of loudness, fluctuation strength, spectral entropy, and spectral skewness were significant parameters in the equal *L_Aeq_* condition, while ϕ_1_, τ_e_, roughness, fluctuation strength, and the QR of spectral entropy were significant parameters in the equal loudness condition:

*SV*_insect song in equal SPL_ ≈ i_0_ + i_1_ × W_ϕ(0)__QR+ i_2_ × loudness_QR + i_3_ × fluctuation strength + i_4_ × spectral entropy + i_5_ × spectral skewness, (3)

*SV*_insect song in equal loudness_ ≈ j_0_+ j_1_ × ϕ_1_ + j_2_ × τ_e_ + j_3_ × roughness + j_4_ × fluctuation strength + j_5_ × spectral entropy_QR. (4)

The correlation coefficients between all of the explanatory variables in the equal *L_Aeq_* and loudness conditions are shown in Table 4 and Table 5. The ANOVA indicated the statistical significance of the model (*F*(5, 250) = 106.00, *p* < 0.001 for the equal *L_Aeq_* condition; *F*(5, 234) = 14.58, *p* < 0.001 for the equal loudness condition). The adjusted coefficient of determination, R^2^, was 0.68 for the equal *L_Aeq_* condition and 0.22 for the equal loudness condition. The standardized partial regression coefficients in Equations (3) and (4) are summarized in Table 6.

Fluctuation strength was a significant predictive variable in both the equal *L_Aeq_* and loudness conditions. This suggests that strong and slow amplitude modulation of insect songs is important for salience perception. Although IACC was a significant predictor of birdsong salience in both the equal *L_Aeq_* and loudness conditions, it was not a significant predictor of insect song salience. This may be because the insect songs used in the experiment were not recorded using a dummy head microphone [1], and so spatial impressions of the sound sources were not accurately reproduced.

Loudness variations can also be important for saliency. The QR of loudness was a significant predictive variable in the equal loudness condition. This is consistent with the results for birdsongs in the equal *L_Aeq_* condition, although the partial regression coefficient was negative for insect songs and positive for birdsongs. This suggests that sound sources with moderate loudness variations are perceived to be more salient. We observed a similar pattern for roughness. Roughness was a significant predictor of both birdsong and insect song salience in the equal loudness condition. Although the partial regression coefficient for birdsong was positive, that for insect songs was negative. This suggests that sound sources with moderately fast amplitude modulation are perceived to be more salient.

Spectral entropy appears to play an important role in saliency. Spectral entropy and the QR of spectral entropy were significant predictors of salience in the equal *L_Aeq_* and loudness conditions, respectively. The positive partial regression coefficient of spectral entropy suggests that abrupt energy changes in the frequency domain of a sound increase the salience. This is partially consistent with our finding regarding the role of spectral flux in the salience of birdsongs. The negative partial regression coefficient of the QR of spectral entropy suggests that stable energy changes in the frequency domain are more important for saliency.

Pitch strength can also be an important modulator of saliency. The maximum peak amplitude of the ACF, ϕ_1_, was a significant predictor of insect song salience in the equal loudness condition. The negative partial regression coefficient of ϕ_1_ suggests that broader frequency components are necessary for salience. This is inconsistent with previous findings regarding preference [1]. One possible explanation for this discrepancy is the importance of tonal components for preference, specifically, the importance of melody and broader frequency components for salience, as they enable the listener to more deeply understand the characteristics of the sound source.

## 4. Conclusions

We examined the salience of birdsong and insect song in terms of several physical parameters. The results indicated that Horornis diphone and Cuculus canorus produce the most salient birdsongs, while Meimuna opalifera and Oncotympana maculaticollis produce the most salient insect songs. All of these creatures are well-known in Japan. The variation of loudness, roughness, and spectral skewness were significant predictors of salience for both birdsongs and insect songs. Spatial content related to the interaural cross-correlation coefficient, IACC, and spectral content expressed by spectral flux were significantly associated with birdsong salience. The maximum peak amplitude of the ACF, ϕ_1_, was significantly associated with insect song salience. These findings may be useful to designers of sound landmarks regarding physical parameters to consider, such as ϕ_1_, IACC, and spectral skewness.

Considering the findings of the current study together with those of a previous study on preference for birdsongs [1], the birdsongs of Horornis diphone and Cuculus canorus appear to be desirable information signals because they are salient and preferred. As for insect songs, the song of Tanna japonensis appears to be a desirable signal because it is salient and preferred. This may be partly because they are ubiquitous in Japan, where they are well-liked.

Subjective salience in the current study was not well correlated with the physical parameters of the sounds. Soundscapes are not only affected by the physical aspects of sounds, but also by the context, which includes relationships between people and activities and position in space and time. Thus, the context with respect to the participants may be a dominant factor influencing subjective salience, and could be an interesting topic for future study. Furthermore, the salience of birdsong and insect song stimuli may differ according to culture. We hope to examine cognitive and cultural factors influencing salience in future work. In addition, the present findings need to be verified in a study with visually impaired participants.

## Figures and Tables

**Table 1:** The correlation coefficients between the explanatory variables used in the multiple regression analysis in the equal *L_Aeq_* condition for the birdsongs.

	Entropy	Spectral Flux	Spectral Skewness
IACC	0.44	−0.49	0.17
Entropy		−0.31	0.43
Spectral flux			−0.49

**Table 2:** The correlation coefficients between the explanatory variables used in the multiple regression analysis in the equal loudness condition for the birdsongs.

	τ_ε__QR	W_ϕ(0)__QR	IACC	Loudness_QR	Roughness
_τ1_	0.51	0.40	0.16	0.01	−0.30
τ_ε__QR		−0.04	0.25	0.01	−0.19
W_ϕ(0)__QR			−0.61	0.22	−0.54
IACC				−0.35	0.43
Loudness_QR					−0.22

**Table 3:** Significant predictive parameters and standardized partial regression coefficients revealed by multiple linear regression analyses of birdsong salience.

	Predictive Parameter	Standardized Partial Regression Coefficients
Equal *L_Aeq_*	b_1_, IACC **	0.18
	b_2_, entropy *	−0.15
	b_3_, spectral flux **	0.38
	b_4_, spectral skewness **	0.79
Equal loudness	c_1_, τ_1_ **	−0.57
	c_2_, τ_e__QR **	0.25
	c_3_, W_ϕ(0)__QR **	0.37
	c_4_, IACC **	0.36
	c_5_, loudness_QR **	0.49
	c_6_, roughness **	0.17

Asterisks indicate the level of significance, i.e., ** *p* < 0.01, * *p* < 0.05.

**Table 4:** The correlation coefficients between the explanatory variables used in the multiple regression analysis in the equal *L_Aeq_* condition for insect songs.

	Loudness_QR	Fluctuation Strength	Spectral Entropy	Spectral Skewness
W_ϕ(0)__QR	0.31	0.38	0.18	0.08
Loudness_QR		−0.05	0.01	−0.44
Fluctuation strength			0.23	0.27
Spectral entropy				0.32

**Table 5:** The correlation coefficients between the explanatory variables used in the multiple regression analysis in the equal loudness condition for insect songs.

	τ_e_	Roughness	Fluctuation Strength	Spectral Entropy_QR
ϕ_1_	0.55	0.18	0.59	−0.19
τ_e_		0.10	0.46	−0.20
Roughness			0.03	−0.11
Fluctuation strength				0.01

**Table 6:** Significant predictive parameters and standardized partial regression coefficients revealed by multiple linear regression analyses of inset song salience.

	Predictive Parameter	Standardized Partial Regression Coefficients
Equal *L_Aeq_*	i_1_, W_ϕ(0)__QR *	0.15
	i_2_, loudness_QR **	−0.25
	i_3_, fluctuation strength **	0.36
	i_4_, spectral entropy **	0.44
	i_5_, spectral skewness **	0.18
Equal loudness	j_1_, ϕ_1_ **	−0.35
	j_2_, τ_e_ **	0.24
	j_3_, roughness **	−0.32
	j_4_, fluctuation strength **	0.20
	j_5_, spectral entropy_QR **	−0.24

Asterisks indicate the level of significance, i.e., ** *p* < 0.01, * *p* < 0.05.
